# Rheumatoid Meningitis Occurring during Etanercept Treatment

**DOI:** 10.1155/2017/7638539

**Published:** 2017-02-13

**Authors:** Koji Tsuzaki, Takashi Nakamura, Hiroyuki Okumura, Naoko Tachibana, Toshiaki Hamano

**Affiliations:** ^1^Department of Neurology, Kansai Electric Power Hospital, Osaka, Japan; ^2^Department of Neurology, National Cerebral and Cardiovascular Center, Osaka, Japan; ^3^Okumura Clinic, Shiga, Japan

## Abstract

We report a 65-year-old man who had repetitive seizures 6 months after receiving etanercept, methotrexate, and prednisolone for rheumatoid arthritis. Mononuclear cells were mildly increased in the cerebrospinal fluid (CSF). Brain magnetic resonance imaging (MRI) showed high intensity along sulci of the frontal and parietal lobes. Brain biopsy revealed lymphocyte and plasma cell infiltration in the meninges, confirming the diagnosis of rheumatoid meningitis. After steroid pulse therapy, seizures resolved and clinical findings improved. When etanercept was replaced by tocilizumab, rheumatoid meningitis did not recur. Although TNF-*α* inhibitors can control joint symptoms of rheumatoid arthritis, they may induce rheumatoid meningitis.

## 1. Introduction

The common neurological complications of rheumatoid arthritis are peripheral neuropathies, such as carpal tunnel syndrome and myelopathy due to atlantoaxial subluxation. It is very rare to find complications involving pathologies inside the cranium. In the central nervous system, rheumatoid arthritis targets the meninges, resulting in rheumatoid meningitis [1]. We report a case of rheumatoid meningitis that occurred during etanercept and methotrexate treatment.

## 2. Case Report

**Figure 1:**
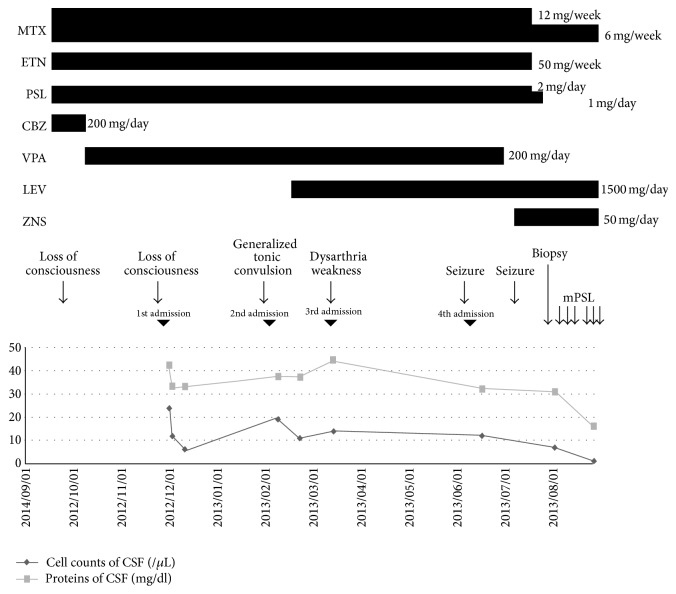
Clinical course. After the treatment with intravenous methylprednisolone, cell counts and proteins of CSF decreased, and seizures disappeared. MTX: methotrexate, ETN: etanercept, PSL: prednisolone, CBZ: carbamazepine, VPA: sodium valproate, LEV: levetiracetam, ZNS: zonisamide, and mPSL: methylprednisolone.

A 65-year-old man came to our clinic in September 2012 with a complaint of transient loss of consciousness. His medical history included prostatomegaly, hypertension, and idiopathic thrombocytopenic purpura. He had been diagnosed as having rheumatoid arthritis in February 2012, which was well controlled by methotrexate (12 mg/week), etanercept (50 mg/week), and prednisolone (2 mg/day). There was no notable family history.  Figure 1 shows clinical course (Figure 1).

He was neurologically intact. There were no abnormal findings on brain computerized tomography (CT) and MRI. Electroencephalography (EEG) showed intermittent bursts of bilateral delta activity and infrequent high-amplitude sharp waves predominantly in the frontal regions. Carbamazepine (200 mg/day) was administered for suspected epilepsy, which was later changed to sodium valproate (200 mg/day) due to the occurrence of rash.

After this episode, he showed three transient episodes such as loss of consciousness, generalized tonic convulsion followed by consciousness disturbance, and dysarthria associated with left leg weakness during the following 6 months. Each episode led to hospital admission, but the patient had no neurological symptoms when he was investigated. Brain MRI showed abnormal signals and contrast enhancement in sulci of the left frontal and parietal lobes, but no definite epileptiform activity was found on EEG. CSF revealed nonspecific mild pleocytosis. At this point, chronic meningitis was diagnosed, but the cause was not confirmed.

He was admitted for the fourth time in June 2013, because of a seizure continuing for several minutes and persistent disturbance of consciousness. The patient was slightly disorientated. Examinations of the cranial nerves and motor and sensory systems and deep tendon reflexes and coordination were normal. Serological examination showed that blood platelet count was decreased (58000/*μ*L), without elevated white blood cell count. On biochemical examination, hepatic enzymes and kidney function were found to be normal, and there were no findings suggesting inflammation. CK was mildly elevated (346 IU/L). Tumor markers were all normal, and sIL2R was mildly elevated at 555 U/mL (reference range: 145–519). Immunological examination showed 12 IU/mL for RF (normal range: 0–10), 80 times for ANA (normal range: 0–40), 297 U/mL for SS-A antibodies (normal range: 0–10), and 18.6 U/mL for SS-B antibodies (normal range: 0–10). PR3-ANCA and MPO-ANCA were normal, and anti-CCP antibodies were 275 U/mL (normal range: 0–4.5). CSF examination showed cell count of 12/*μ*L (mononuclear cells: 12/*μ*L), protein levels of 32 mg/dL, and glucose levels of 55 mg/dL (blood glucose 70 mg/dL). IgG index was 1.7, and cytodiagnosis was negative. CSF culture and PCR for acid-fast bacterium were negative.

**Figure 2:**
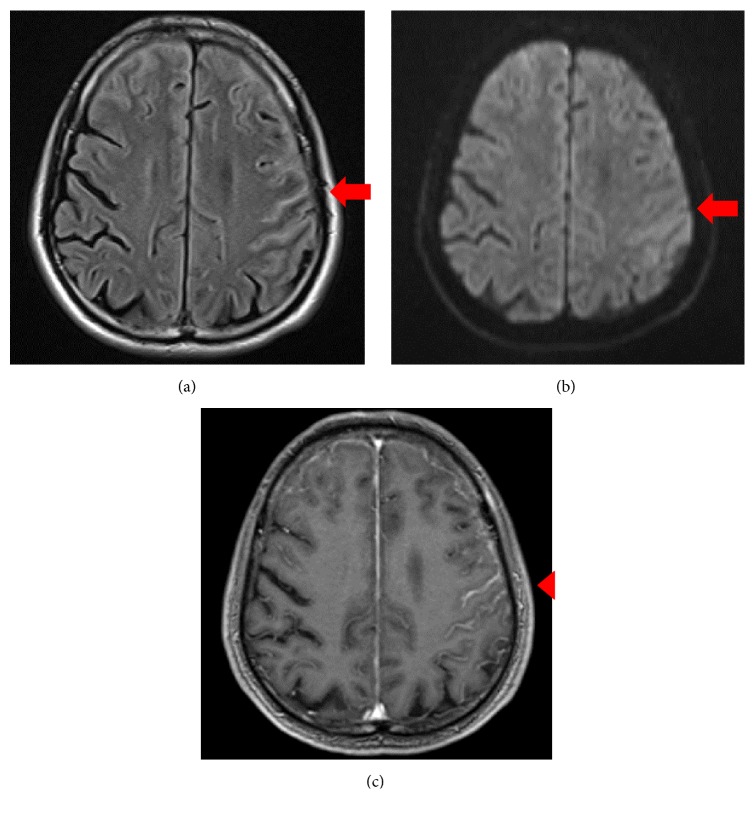
Brain MRI findings on the 4th admission. (a) Axial T2 fluid-attenuated inversion recovery (FLAIR) image (1.5 T; TR 10000 ms, TE 108 ms). (b) Diffusion-weighted image (DWI) (1.5 T; TR 5000 ms, TE 75 ms). (c) T1-weighted image (T1WI) enhanced by gadolinium (1.5 T; TR 7.50 ms, TE 2.82 ms). MRI shows extensive thick linear hyperintensity of the leptomeninges in bilateral frontal lobe and the left parietal lobe on T2 FLAIR and DWI (red arrows). T1WI after gadolinium administration shows enhancement of the leptomeninges (red arrowhead).

Brain MRI showed high intensity areas mainly in the left paracentral sulcus and bilateral superior frontal sulcus in fluid-attenuated inversion recovery and diffusion-weighted imaging scans. Contrast enhancement was observed in the left paracentral sulcus and bilateral superior frontal sulcus. Although the cortex of the left parietal lobe had mild swelling, there were no abnormal findings in the white matter (Figure 2). On EEG, bilateral high-amplitude frontal delta wave bursts of 3–5 Hz occurred in the frontal regions with no epileptiform discharges.

The patient's level of consciousness was improved after hospitalization. Because his rheumatoid arthritis was well controlled and there was thrombocytopenia, methotrexate was reduced to 6 mg/week, and prednisolone and etanercept were discontinued prior to brain biopsy. Zonisamide (50 mg/day) was substituted for sodium valproate, because the patient had another seizure lasting for one hour. New findings on brain CT, MRI, or EEG were not observed at that time.

**Figure 3:**
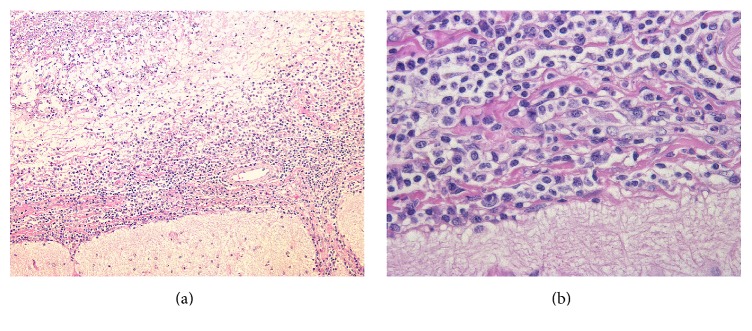
Brain biopsy. Microscopic view of biopsied specimen showed leptomeningitis with plasma cells and lymphocytes infiltration. Haematoxylin and eosin stain, magnification of (a) 100x and (b) 400x.

A brain biopsy from the lesion in the right frontal lobe showed invasion of lymphocytes and plasma cells into the superficial brain layers, resulting in necrosis of the brain surface (Figure 3). The lymphocytes were small; an enzyme antibody technique showed that they were a mixture of CD3- and CD20-positive cells, and light chain restriction was not apparent for plasma cells, indicating that they were unlikely to be tumorous. Flow cytometry also did not show tumor patterns. There were no findings of vasculitis. No fungi or acid-fast bacterium were observed.

**Figure 4:**
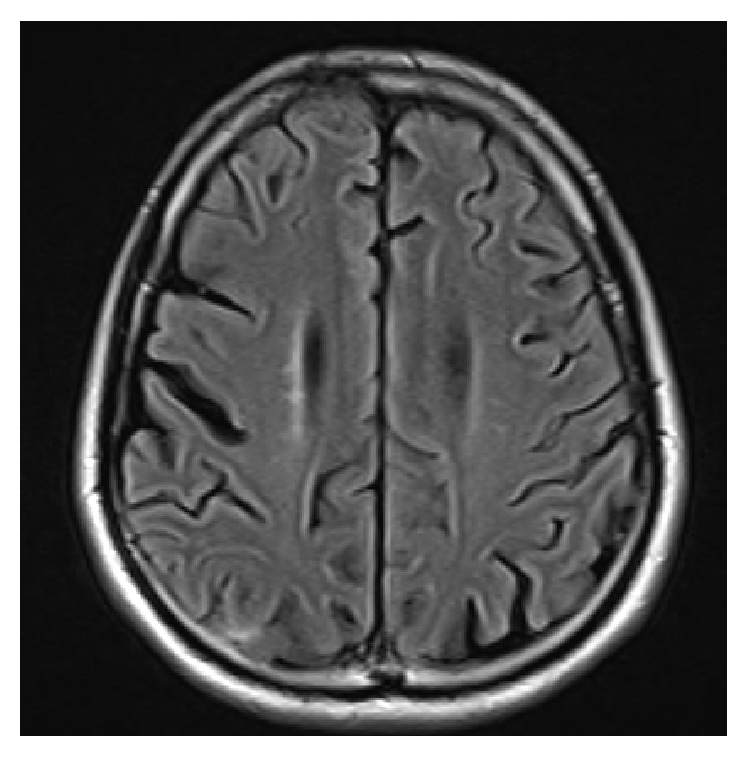
Brain MRI finding 2 years after treatment. T2 FLAIR image (1.5 T; TR 10000 ms, TE 108 ms). MRI performed 2 years after treatment no longer shows linear hyperintensity of the leptomeninges.

Based on the pathological findings, we diagnosed the patient as having rheumatoid meningitis and performed 2 courses of steroid pulse therapy (methylprednisolone 1000 mg/day × 3 days). After the steroid pulse therapy, seizures disappeared and cell counts in the CSF returned to normal. On brain MRI, abnormal signals in the sulci improved (Figure 4), and the slow waves were rarely observed on EEG. In terms of treatment for rheumatoid arthritis, joint symptoms appeared when using methotrexate alone. We added tocilizumab, an IL-6 receptor antagonist, starting in May 2014, and joint symptoms have been well controlled since then. There has also been no recurrence of seizures during the 2 years following the steroid pulse therapy.

## 3. Discussion

There are no specific biomarkers for rheumatoid meningitis, and brain MRI and biopsy are essential for its diagnosis [2]. On brain MRI, the meninges show contrast enhancement [2, 3]. Pathologically, invasion of lymphocytes in the meninges, vasculitis, and rheumatoid nodules are the typical characteristics [2, 4]. However, it is rare to find all of these characteristics simultaneously [4]. Although there is no consensus for the treatment of rheumatoid meningitis, steroids are often used as the first choice [2, 5]. Our patient also responded well to steroid treatment. When steroid is not effective enough, additional treatment with cyclophosphamide [6], azathioprine [7], cyclosporine [8], and methotrexate [7] may be useful.

To our knowledge, there have been only four case reports of rheumatoid meningitis during treatment with TNF-*α* inhibitors (Table 1). Huys et al. reported a 58-year-old woman who presented with headache and epilepsy while she was taking methotrexate and adalimumab for rheumatoid arthritis. The meningitis of this patient improved after discontinuation of methotrexate and adalimumab, steroid pulse therapy, and additional administration of rituximab [9]. Ahmed et al. reported a 77-year-old man who had been treated with methotrexate for rheumatoid arthritis. The patient experienced headache, disturbance in consciousness, involuntary movements of the upper and lower limbs, and motor aphasia after adalimumab was added. The symptoms improved after administration of prednisolone, and there was no recurrence after discontinuing adalimumab [5]. Chou et al. reported a 58-year-old woman who presented with headache, slurred speech, numbness of the left side of the face, weakness in the limbs, and seizures. Although her rheumatoid meningitis improved after administration of cyclophosphamide and prednisolone, the symptoms of rheumatoid arthritis deteriorated after discontinuation of cyclophosphamide and reduction of prednisolone. When infliximab was administered, rheumatoid meningitis relapsed. The rheumatoid meningitis improved after discontinuation of infliximab and restarting of cyclophosphamide and prednisolone [6]. Schmid et al. reported a 64-year-old male treated with methotrexate and infliximab. The patient experienced a focal seizure on the right side of the body and aphasia and consciousness disturbance. Symptoms improved after discontinuation of infliximab and steroid pulse therapy [10]. All the patients, including ours, presented with rheumatoid meningitis 2 weeks to 7 months after commencing TNF-*α* inhibitor treatment, which improved with discontinuation of the treatment and steroid pulse therapy. It is possible that TNF-*α* inhibitors induce rheumatoid meningitis. It has been pointed out that TNF-*α* inhibitors can create rheumatoid nodules in a variety of tissues, probably through multiple mechanisms, including modifications of the expression of other cytokines [9, 11]. Another possible explanation is low permeability of etanercept into the brain through the blood-brain barrier [6]. It is possible that etanercept could not suppress the meningitis although it could control the arthritis.

Furthermore, it is widely known that methotrexate can cause aseptic meningitis, particularly in intrathecal administration [12]. In the present patient, methotrexate treatment was reduced prior to biopsy, and there was no recurrence of meningitis after its reduction. Thus, we cannot exclude the possibility that the meningitis was induced by a high dose of methotrexate.

Even when the rheumatoid arthritis is well controlled by TNF-*α* inhibitors, if seizures and disruption in consciousness occur, biopsy and steroid therapy should be considered immediately, as it is possible that the TNF- *α* inhibitors can induce rheumatoid meningitis.

## Figures and Tables

**Table 1:** Cases of rheumatoid meningitis during treatment with a TNF-*α* blocker.

Case (age, sex)	Symptoms	TNF-*α* inhibitor	Duration from administration of TNF-*α* inhibitor to the onset of rheumatoid meningitis	Treatment
58F [9]	Headache, psychomotor retardation, focal seizures	Adalimumab	7 months	Discontinuation of adalimumab Steroid Rituximab

77M [5]	Headache, expressive dysphasia, involuntary movements of upper extremities, confusion	Adalimumab	2 weeks	Discontinuation of adalimumab Steroid

58F [6]	Headache, emotional lability, left facial numbness, slurred speech, weakness and numbness of the extremities, frequent falls, seizures	Infliximab	3 months	Discontinuation of infliximab Steroid Cyclophosphamide

64M [10]	Aphasia, convulsion, focal seizure of the right side of the body	Infliximab	7 months	Discontinuation of infliximab Steroid

65M (present case)	Seizure	Etanercept	6 months	Discontinuation of etanercept Steroid
